# Improving Genomic Prediction Using High-Dimensional Secondary Phenotypes

**DOI:** 10.3389/fgene.2021.667358

**Published:** 2021-05-24

**Authors:** Bader Arouisse, Tom P. J. M. Theeuwen, Fred A. van Eeuwijk, Willem Kruijer

**Affiliations:** ^1^Biometris, Wageningen University and Research, Wageningen, Netherlands; ^2^Laboratory of Genetics, Wageningen University and Research, Wageningen, Netherlands

**Keywords:** GBLUP, genomic prediction, secondary traits, selection indices, penalized regression, random forest

## Abstract

In the past decades, genomic prediction has had a large impact on plant breeding. Given the current advances of high-throughput phenotyping and sequencing technologies, it is increasingly common to observe a large number of traits, in addition to the target trait of interest. This raises the important question whether these additional or “secondary” traits can be used to improve genomic prediction for the target trait. With only a small number of secondary traits, this is known to be the case, given sufficiently high heritabilities and genetic correlations. Here we focus on the more challenging situation with a large number of secondary traits, which is increasingly common since the arrival of high-throughput phenotyping. In this case, secondary traits are usually incorporated through additional relatedness matrices. This approach is however infeasible when secondary traits are not measured on the test set, and cannot distinguish between genetic and non-genetic correlations. An alternative direction is to extend the classical selection indices using penalized regression. So far, penalized selection indices have not been applied in a genomic prediction setting, and require plot-level data in order to reliably estimate genetic correlations. Here we aim to overcome these limitations, using two novel approaches. Our first approach relies on a dimension reduction of the secondary traits, using either penalized regression or random forests (LS-BLUP/RF-BLUP). We then compute the bivariate GBLUP with the dimension reduction as secondary trait. For simulated data (with available plot-level data), we also use bivariate GBLUP with the penalized selection index as secondary trait (SI-BLUP). In our second approach (GM-BLUP), we follow existing multi-kernel methods but replace secondary traits by their genomic predictions, with the advantage that genomic prediction is also possible when secondary traits are only measured on the training set. For most of our simulated data, SI-BLUP was most accurate, often closely followed by RF-BLUP or LS-BLUP. In real datasets, involving metabolites in Arabidopsis and transcriptomics in maize, no method could substantially improve over univariate prediction when secondary traits were only available on the training set. LS-BLUP and RF-BLUP were most accurate when secondary traits were available also for the test set.

## 1. Introduction

Genomic prediction is increasingly applied as standard tool in many animal and plant breeding programs. Since it was first introduced by Meuwissen et al. (2001), the main objective of genomic prediction was to estimate the breeding values for unphenotyped (test) genotypes with only molecular markers, using a training population for which both phenotypic and genotypic data are available. Applications of genomic prediction facilitate the rapid selection of superior genotypes (genomic selection) and accelerate genetic progress in crop breeding.

At the same time, advances in high-throughput phenotyping and cell biology technologies provide increasing amounts of phenotypic data, in addition to the “primary” or “target” traits of interest, such as yield or disease resistance. Such additional traits are typically high-dimensional, and collected using various types of technology, e.g., remote-sensing (Araus et al., 2018), machine vision (Yang et al., 2020), and automation technology (Sun et al., 2019). Common situations are that secondary traits are measured (1) in the field, on the same plant as the target trait, but much earlier in the growing season (2) on entirely different plants, in controlled environments in phenotyping platforms. In both cases, the secondary traits are either observed only for the training set of genotypes, or also for the test set. In all cases however, the question is whether some of the secondary traits are associated with the target traits of interest, and whether these correlations are genetic. In a genomic prediction context, the question becomes when and how secondary traits can improve prediction for the target trait. This is well understood if there is only one secondary trait: accuracy for the target trait then improves when the heritability of the target trait is lower than the heritability of the secondary trait times the squared genetic correlation (Schulthess et al., 2016; Velazco et al., 2019). Here we focus on the more challenging situation with a large numbers of secondary traits, which is increasingly common since the arrival of high-throughput phenotyping.

The two main approaches to incorporate high-dimensional secondary traits in genomic prediction are the use of multiple relatedness matrices, and penalized selection indices. In the former approach, the target trait is modeled as the sum of genetic effects and effects from secondary traits. Both type of effects are random, and the relative importance of these contributions is estimated either using REML-estimates for variance components or cross-validation. Predictions for the test set are the sum of the BLUPs for the different effects. Examples of this approach are Fu et al. (2012), who obtained a high level of accuracy for predicting hybrid yield performance using gene expression data from the hybrid parents. Similarly, Riedelsheimer et al. (2012) reported moderate to high accuracies for yield-related traits using 120 metabolites in maize. Schrag et al. (2018) and Xiang et al. (2019) used different relatedness matrices corresponding to different types of -omics data. Two major limitations of multiple random-effects models are that (1) they cannot be used when secondary traits are only available on the training set; (2) they cannot distinguish between genetic and residual correlations among the target and secondary traits.

The second approach was recently proposed by Lopez-Cruz et al. (2020), who extended classical selection indices by imposing a LASSO or ridge penalty on the coefficients. This achieves a dimension reduction, replacing the secondary traits by a single selection index *S*, which is a linear combination of the original traits. The coefficients are chosen to maximize $h^{2}\left(S\right)\rho_{G}^{2}\left(Y,S\right)$, i.e., the heritability of *S* times the squared genetic correlation between *S* and the target trait (*Y*). Lopez-Cruz et al. (2020) found that on new data, this quantity was indeed much higher than for the classical (unpenalized) selection index. Despite this promising result, penalized selection indices have not yet been applied in a genomic prediction context. One possible reason may be that accurate estimates of genetic correlations between *Y* and each of the secondary traits are required, for which the availability of plant/plot-level observations is assumed.

In the present paper, we propose two new approaches to deal with large numbers of secondary traits, and compare these to the approaches described above, using simulated and real data. First, we define genomic prediction using alternative dimension reductions (LS-BLUP/RF-BLUP), relying on penalized regression (or random forest regression) of the target on the secondary traits. We then compute the bivariate GBLUP with the dimension reduction as secondary trait. Second, we extend existing multi-kernel methods by replacing the secondary traits by their genomic predictions, the main advantage being that genomic prediction for the test set is always possible, also when secondary traits are only measured on the training set. For simulated data (with available plot-level data), we will also use bivariate GBLUP with the penalized selection index as secondary trait (SI-BLUP).

## 2. Materials and Methods

### 2.1. Distributional Assumptions

To a large extent we follow the notation of Runcie and Cheng (2019), assuming observations on traits *Y*_1_, …, *Y*_*p*+1_, where each *Y*_*j*_ is a column vector. The first one (*Y*_1_ = *Y*_*f*_) is the focal or target trait, for which genomic predictions are required; *Y*_2_, …, *Y*_*p*+1_ are the secondary traits. $Y_{s}={\left(Y_{2}^{t},\ldots ,Y_{p+1}^{t}\right)}^{t}$ is the column vector containing all secondary traits; similarly, $Y={\left(Y_{1}^{t},\ldots ,Y_{p+1}^{t}\right)}^{t}$ is the column vector containing all traits. We have in total *n* = *n*_*t*_ + *n*_*o*_ genotypes, including *n*_*o*_ genotypes for which the target trait is observed (the training set), and *n*_*t*_ for which it is to be predicted (the *t* referring to test set). We will use subscripts *t* and *o* to indicate that we take the subset of values on the test, respectively training set, for example *Y*_*o*_ and *Y*_*f, o*_.

The secondary phenotypes are either observed only on the training set (the CV1-scenario, using the terminology of Runcie and Cheng, 2019), or also for the test genotypes (CV2). Since our focus here is on variable selection and dimension reduction (rather than different cross-validation schemes), we will refer to these simply with scenarios 1 and 2, respectively. The *n* × *n* genetic relatedness matrix *K* is partitioned as:

$$\begin{array}{l} K=\left(\begin{array}{ll} K_{tt} & K_{to}\\ K_{ot} & K_{oo} \end{array}\right), \end{array}$$

where the *n*_*t*_ × *n*_*o*_ matrix *K*_*to*_ defines the relatedness between new (test) and observed (training) genotypes. We will also write *K*_*t*·_ = [*K*_*tt*_ *K*_*to*_] and *K*_*o*·_ = [*K*_*ot*_ *K*_*oo*_]. Similarly, we can decompose the genetic and residual covariance matrices Σ^*u*^ and Σ^*e*^ as

$$\begin{array}{l} \Sigma^{u}=\left(\begin{array}{ll} \Sigma_{ff}^{u} & \Sigma_{fs}^{u}\\ \Sigma_{sf}^{u} & \Sigma_{ss}^{u} \end{array}\right)=\left(\begin{array}{c} \Sigma_{f\cdot }^{u}\\ \Sigma_{s\cdot }^{u} \end{array}\right), \end{array}$$

$$\begin{array}{l} \Sigma^{e}=\left(\begin{array}{ll} \Sigma_{ff}^{e} & \Sigma_{fs}^{e}\\ \Sigma_{sf}^{e} & \Sigma_{ss}^{e} \end{array}\right)=\left(\begin{array}{c} \Sigma_{f\cdot }^{e}\\ \Sigma_{s\cdot }^{e} \end{array}\right), \end{array}$$

where the scalars $\Sigma_{ff}^{u}$ and $\Sigma_{ff}^{e}$ are respectively the genetic and residual variance of the focal trait, and the matrices $\Sigma_{ss}^{u}$ and $\Sigma_{ss}^{e}$ contain the genetic and residual (co)variances of the secondary traits. The row-vectors $\Sigma_{fs}^{u}$ and $\Sigma_{fs}^{e}$ contain the genetic and residual covariance between the focal and the secondary traits.

The joint distribution of *Y* = (*Y*_1_, …, *Y*_*p*+1_) is assumed to be

(1) $$\begin{array}{l} \begin{array}{l} Y=X\beta +U+E\\ =\left[\begin{array}{c} Y_{1}\\ \vdots \\ Y_{p+1} \end{array}\right]=\left[\begin{array}{c} X_{1}\beta_{1}\\ \vdots \\ X_{p+1}\beta_{p+1} \end{array}\right]+\left[\begin{array}{c} U_{1}\\ \vdots \\ U_{p+1} \end{array}\right]+\left[\begin{array}{c} E_{1}\\ \vdots \\ E_{p+1} \end{array}\right]\\ =\left[\begin{array}{c} Y_{f}\\ Y_{s} \end{array}\right]=\left[\begin{array}{c} X_{f}\beta_{f}\\ X_{s}\beta_{s} \end{array}\right]+\left[\begin{array}{c} U_{f}\\ U_{s} \end{array}\right]+\left[\begin{array}{c} E_{f}\\ E_{s} \end{array}\right], \end{array} \end{array}$$

where

(2) $$\begin{array}{l} U\sim N\left(0,\Sigma^{u}⊗K\right),\;E\sim N\left(0,\Sigma^{e}⊗I_{n}\right). \end{array}$$

The genetic covariances ($\Sigma_{fs}^{u}$) quantify the degree of overlap among genetic signals, based on which multivariate methods can potentially improve genomic prediction. The residual covariances ($\Sigma_{fs}^{e}$) are important when traits are measured on the same individuals; if measured on different individuals (typically, in a different experiment), Σ^*e*^ can assumed to be diagonal. Σ^*u*^ and Σ^*e*^ are usually unknown, and need to be estimated from the data. For *p* larger than 5 − 10, this usually requires approximations. Below we describe several dimension reduction approaches, which reduce the dimensionality of the secondary phenotypes to 1, and exact REML-estimates of Σ^*u*^ and Σ^*e*^ can be obtained with standard software.

### 2.2. Genomic Prediction

The main objective is the prediction of the genetic effect *U*_1_ = *U*_*f*_, i.e., the breeding values for the focal trait, in particular for the test set (*U*_*f, t*_). In our simulations we assess prediction accuracy in terms of the Pearson correlation (*r*) between the simulated and predicted genetic effects, on the test set. For real data, we consider the correlation between the predicted genetic effects and the trait values observed on the test sets. Although it is well-known that this is a biased estimator of the true accuracy (i.e., the correlation with the unknown genetic effect), the bias is likely to be constant among methods, as long as the target and secondary traits are observed on different plants (Runcie and Cheng, 2019).

### 2.3. Univariate GBLUP

The univariate GBLUP for *U*_*f, t*_ is defined by

(3) $$\begin{array}{l} \begin{array}{l} {\hat{U}}_{f,t}^{\left(\text{uni}\right)}=E\left(U_{f,t}|Y_{f,o}\right)={\hat{\Sigma }}_{ff}^{u}K_{to}{\hat{V}}^{-1}\left(Y_{f,o}-X_{f,o}{\hat{\beta }}_{f}\right)\\ \;=K_{to}K_{oo}^{-1}{\hat{U}}_{f,o}^{\left(\text{uni}\right)},\\ {\hat{U}}_{f,o}^{\left(\text{uni}\right)}={\hat{\Sigma }}_{ff}^{u}K_{oo}{\hat{V}}^{-1}\left(Y_{f,o}-X_{f,o}{\hat{\beta }}_{f}\right),\\ \;\hat{V}={\hat{\Sigma }}_{ff}^{u}K_{oo}+{\hat{\Sigma }}_{ff}^{e}I_{n_{o}}, \end{array} \end{array}$$

where ${\hat{U}}_{f,o}^{\left(\text{uni}\right)}$ is the GBLUP for the training set, and REML-estimates of β_*f*_ and the variance components $\Sigma_{ff}^{u}$ and $\Sigma_{ff}^{e}$ are obtained from a univariate mixed model for *Y*_*f*_. This is the best (univariate) linear unbiased predictor, at least given the true values of the variance components.

### 2.4. Multivariate GBLUP in Scenarios 1 and 2

The multivariate GBLUP in scenario 1 is

(4) $$\begin{array}{l} \begin{array}{l} {\hat{U}}_{f,t}^{\left(m1\right)}=E\left(U_{f,t}|Y_{o}\right)=\left({\hat{\Sigma }}_{f\cdot }^{u}⊗K_{to}\right){\hat{V}}^{-1}\left(Y_{o}-X_{o}\hat{\beta }\right)\\ \;=K_{to}K_{oo}^{-1}{\hat{U}}_{f,o}^{\left(m1\right)},\\ {\hat{U}}_{f,o}^{\left(m1\right)}=\left({\hat{\Sigma }}_{f\cdot }^{u}⊗K_{oo}\right){\hat{V}}^{-1}\left(Y_{o}-X_{o}\hat{\beta }\right),\\ \;\hat{V}={\hat{\Sigma }}^{u}⊗K_{oo}+{\hat{\Sigma }}^{e}⊗I_{n_{o}}, \end{array} \end{array}$$

where ${\hat{U}}_{f,o}^{\left(m1\right)}$ is the GBLUP for the training set, and REML-estimates of β and the variance components (matrices) Σ^*u*^ and Σ^*e*^ are obtained from the multivariate mixed model for *Y*_*f*_ and *Y*_*s*_. As pointed out by Runcie and Cheng (2019), ${\hat{U}}_{f,t}^{\left(m1\right)}$ and ${\hat{U}}_{f,t}^{\left(\text{uni}\right)}$ have the same form, but the “input” Û_*f, o*_ differs.

The multivariate GBLUP in scenario 2 is

(5) $$\begin{array}{l} {\hat{U}}_{f,t}^{\left(m2\right)}=E(U_{f,t}|Y_{f,o},Y_{s})\\ \;=\left(\begin{array}{cc} {\hat{\Sigma }}_{ff}^{u}⊗K_{to} & {\hat{\Sigma }}_{fs}^{u}⊗K_{t\cdot } \end{array}\right)\;{\hat{V}}^{-1}\left(\begin{array}{c} Y_{f,o}-X_{f,o}{\hat{\beta }}_{f}\\ Y_{s}-X_{s}{\hat{\beta }}_{s} \end{array}\right)\text{​},\\ \;\hat{V}=\left(\begin{array}{cc} {\hat{\Sigma }}_{ff}^{u}K_{oo} & {\hat{\Sigma }}_{fs}^{u}⊗K_{o\cdot }\\ {\hat{\Sigma }}_{sf}^{u}⊗K_{o\cdot }^{t} & {\hat{\Sigma }}_{ss}^{u}⊗K \end{array}\right)\\ \;+\left(\begin{array}{cc} {\hat{\Sigma }}_{ff}^{e}I_{n_{o}} & {\hat{\Sigma }}_{fs}^{e}⊗\left(\begin{array}{cc} 0 & I_{n_{o}} \end{array}\right)\\ {\hat{\Sigma }}_{sf}^{e}⊗\left(\begin{array}{c} 0^{t}\\ I_{n_{o}} \end{array}\right) & {\hat{\Sigma }}_{ss}^{e}⊗I_{n} \end{array}\right) \end{array}$$

where 0 denotes a *n*_*t*_ × *n*_*o*_ matrix of zeros. This differs from the CV2 prediction in Runcie and Cheng (2019), who described a two-step approach.

### 2.5. Dimension Reduction Using LASSO or Random Forests

Expressions (4) and (5) are valid regardless whether there is just a single secondary phenotype, or multiple ones. However, when the dimension of the secondary phenotype (*p*) is larger than 5 − 10, estimation of the required genetic covariances quickly becomes challenging and often infeasible (Zhou and Stephens, 2014; Zwiernik et al., 2017). Moreover, even if estimates of genetic covariance are available, the resulting predictions may be prone to overfitting. Reducing the dimension of the secondary phenotype appears to be a relevant strategy to deal with these issues.

**Figure 1:**
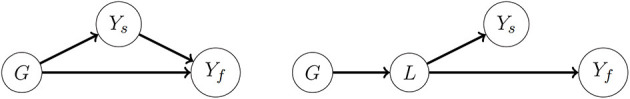
Causal diagrams showing different assumptions about the mechanisms underlying genetic correlations between a high-dimensional secondary phenotype *Y*_*s*_ and a target (focal) trait *Y*_*f*_. For ease of presentation, *Y*_*s*_ is represented by a single node; causal relationships among some of the secondary traits might exist. Outgoing arrows from the node *G* to a trait represent the genetic effect of all loci combined. The arrow *Y*_*s*_ → *Y*_*f*_ represents a causal effect from at least one of the secondary traits on the target trait. **(Left)** Some of the genetic correlations between *Y*_*s*_ and *Y*_*f*_ are the result of the causal effect *Y*_*s*_ → *Y*_*f*_; to some extent they may also be a consequence from correlation between the direct genetic effects *G* → *Y*_*f*_ and *G* → *Y*_*s*_ (see Kruijer et al., 2020 for more mathematical details). **(Right)** There is no causal effect *Y*_*s*_ → *Y*_*f*_, and genetic correlations between them may be induced by genetic effects on a latent trait *L* that is affecting both *Y*_*s*_ and *Y*_*f*_. The **LS-BLUP** and **RF-BLUP** methods assume the left diagram, and reduce the dimension of *Y*_*s*_ first making a prediction Ŷ_*f*_ using *Y*_*s*_ within the training set. Also the **GM-BLUP** method implicitly assumes the left diagram.

Here we propose the dimension reduction *S* = *ĥ*(*Y*_*s*_), where *ĥ*(*Y*_*s*_) is a prediction of *Y*_*f*_ based on *Y*_*s*_, obtained either with LASSO or random forests. Genomic prediction in scenarios 1 and 2 is then performed using (4) and (5), with *S* = *ĥ*(*Y*_*s*_) as secondary trait. We will refer to the resulting genomic predictions using LS-BLUP and RF-BLUP, depending on whether the dimension reduction was achieved by respectively LASSO or random forests. In a GWAS context, such dimension reductions have been used by van Heerwaarden et al. (2015) and Melandri (2019). The intuition behind this dimension reduction is that some of the secondary traits may have a causal effect on *Y*_*f*_ (Figure 1, left). Genomic prediction with LS-BLUP and RF-BLUP may then work well if Ŷ_*f*_ captures most of the relevant genetic correlations. In our simulations described below, we also consider the situation where genetic correlations are not the result of a causal effect of *Y*_*s*_ on *Y*_*f*_ (for example, as in Figure 1, right panel). Because of the relatively small size of the populations considered here, the dimension reduction is computed on the same training set that is used for genomic prediction. This is of course not essential for this approach, and various sample splitting techniques may be of interest for larger populations; see the discussion section below.

When using RF-BLUP in the simulations described below, we used the R-package randomForest, with the default settings. Often however, a more accurate dimension reduction can be achieved by tuning various hyperparameters (like the number of trees), which we explore for the real data.

### 2.6. Dimension Reduction Using Selection Indices

In addition to the notation *Y*_*s*_ for the column vector containing all secondary traits, we will now also use *Y*_*s*_(*j*) for the column-vector containing the *j*th secondary trait, the dimension being either *n*_*o*_ × 1 (scenario 1) or *n* × 1 (scenario 2). We will use $Y_{s}^{\left(i\right)}$ for the row-vector containing all secondary traits for genotype *i*. Recall that the individual secondary traits are still labeled *Y*_2_, …, *Y*_*p*+1_, *Y*_1_ being the target trait.

A well-known alternative dimension reduction approach is to use a selection index $S=\overset{p}{\underset{j=1}{\sum }}\gamma_{j}Y_{s}\left(j\right)$, which is a linear combination of secondary traits, with coefficients such that the resulting index best predicts the genetic effect of the target trait (Falconer and Mackay, 1996). Assuming independent genetic effects (i.e., ignoring population structure), the *p* × 1 vector γ of coefficients is obtained by minimizing, for each individual *i*, the expectation of ${\left(U_{f}\left[i\right]-Y_{s}^{\left(i\right)}\gamma \right)}^{2}$. The minimizing γ then equals the inverse variance-covariance of *Y*_*s*_ times the vector of genetic covariances between *Y*_*s*_ and *Y*_*f*_, i.e., $\gamma^{SI}={\Sigma_{s}}^{-1}\Sigma_{sf}^{u}$. To estimate γ^*SI*^ one could plug in estimates ${\hat{\Sigma }}_{s}$ and ${\hat{\Sigma }}_{sf}^{u}$, where ${\hat{\Sigma }}_{s}={\hat{\Sigma }}_{ss}^{u}⊗K_{oo}+{\hat{\Sigma }}_{ss}^{e}⊗I_{n_{o}}$ is the estimated variance-covariance matrix of the secondary traits on the training population, and ${\hat{\Sigma }}_{sf}^{u}$ contains estimates of genetic covariances with the target trait. However, when the dimension (*p*) is large, $\Sigma_{ss}^{u}$ and $\Sigma_{ss}^{e}$ are difficult to estimate, and the selection index is likely to overfit, as some elements in $\Sigma_{sf}^{u}$ may be large by chance, and receive too much weight.

To address these issues, Lopez-Cruz et al. (2020) proposed penalized selection indices, minimizing instead $E{\left(U_{f}\left[i\right]-Y_{s}^{\left(i\right)}\gamma \right)}^{2}+\lambda J\left(\gamma \right)$, where λ > 0 is the penalty and *J*(γ) is either $\overset{p}{\underset{j=1}{\sum }}\gamma_{j}^{2}$ (ridge penalty) or $\overset{p}{\underset{j=1}{\sum }}|\gamma_{j}|$ (LASSO penalty). λ = 0 gives the classical (unpenalized) SI. In case of a ridge penalty, the penalized SI is given by

(6) $$\begin{array}{l} {\hat{\gamma }}^{SI}\left(\lambda \right)={\left({\hat{\Sigma }}_{s}+\lambda I_{p}\right)}^{-1}{\hat{\Sigma }}_{sf}^{u}. \end{array}$$

We will follow the implementation by Lopez-Cruz et al. (2020) in their R-package SFSI, where $\Sigma_{sf}^{u}$ is estimated with MANOVA on the individual plant or plot-level data, and $\Sigma_{ss}^{u}$ is estimated using the sample covariance matrix of the secondary traits. We emphasize that no multi-trait mixed-model of the form (1)–(2) is fitted. Moreover, the regularization only controls how ${\hat{\Sigma }}_{s}$ affects ${\hat{\Sigma }}_{sf}^{u}$; the estimates ${\hat{\Sigma }}_{s}$ and ${\hat{\Sigma }}_{sf}^{u}$ themselves are not regularized.

Following again (Lopez-Cruz et al., 2020), we use internal cross-validation within the training set to choose an appropriate value of λ, maximizing *h*(*S*)ρ_*G*_(*S, Y*_*f*_). After selecting a value for λ, genomic prediction in scenarios 1 and 2 is performed using (4) and (5), with a single secondary trait, i.e., the selection index $\overset{p}{\underset{j=1}{\sum }}\gamma_{j}^{\left(\lambda \right)}Y_{s}\left[j\right]$. We will use SI-BLUP to refer to the genomic prediction obtained this way.

### 2.7. Genomic Prediction Using Multiple Relatedness Matrices

Another alternative to selection indices is to model the secondary traits using random effects (see e.g., Riedelsheimer et al., 2012; Van De Wiel et al., 2016; Xu et al., 2016; Schrag et al., 2018; Xiang et al., 2019; Azodi et al., 2020). In addition to the genetic relatedness matrix *K*, these models use an additional relatedness matrix *M* derived from the secondary phenotypes, and assume that

(7) $$\begin{array}{l} Y_{f}=X_{f}\beta_{f}+U_{f}^{\left(\text{gen}\right)}+V_{f}^{\left(\text{sec}\right)}+E_{f}=X_{f}\beta_{f}+U_{f}^{\left(\text{gen}\right)}+Y_{s}b_{s}+E_{f}, \end{array}$$

where $U_{f}^{\left(\text{gen}\right)}\sim N\left(0,\sigma_{K}^{2}K\right)$ and $V_{f}^{\left(\text{sec}\right)}\sim N\left(0,\sigma_{M}^{2}M\right)$. We will call this the Multi-BLUP model (not to be confused with Speed and Balding, 2014, where the same type of model is used, but where genomic regions are represented by different relatedness matrices). The variance components $\sigma_{K}^{2}$, $\sigma_{M}^{2}$, and $\sigma_{E}^{2}$ can be estimated with REML or with cross-validation. For simplicity we consider only one type of secondary phenotypes. Similar to the equivalence between GBLUP and SNP-BLUP, the effects $V_{f}^{\left(\text{sec}\right)}$ can be written as *Y*_*s*_*b*_*s*_, for a vector *b*_*s*_ of independent random effects with $N\left(0,p^{-1}\sigma_{M}^{2}\right)$ distribution. Hence, similar to the LS-BLUP and RF-BLUP, the Multi-BLUP approach implicitly assumes a causal effect of *Y*_*s*_ on *Y*_*f*_ (Figure 1, left), which is assumed to be linear, with random coefficients. The usual “genomic” prediction based on model (7) is

(8) $$\begin{array}{l} {\hat{U}}_{\text{Multi}}={\hat{U}}_{f}^{\left(\text{gen}\right)}+{\hat{V}}_{f}^{\left(\text{sec}\right)}, \end{array}$$

i.e., the sum of the BLUPs for the genetic and secondary trait effects. We put genomic between quotes because (8) is partly a phenotypic prediction: instead of the genetic component of the secondary traits, it directly relies on these traits themselves, which are assumed to be available on the test set. As a consequence, the use of (8) is limited to scenario 2.

To overcome these limitations we propose the GM-BLUP:

(9) $$\begin{array}{l} {\hat{U}}_{GM}={\hat{U}}_{f}^{\left(\text{gen}\right)}+{\hat{U}}_{s}^{\left(\text{gen}\right)}{\hat{b}}_{s}, \end{array}$$

where ${\hat{b}}_{s}$ is the vector of predicted random coefficients obtained from the Multi-BLUP model, and ${\hat{U}}_{s}^{\left(\text{gen}\right)}$ is the matrix of GBLUPs for the secondary traits (either univariate or multivariate). These GBLUPs can of course also be computed in scenario 1. Apart from being the “genomic analogue” of (8), (9) can also be motivated by a causal model of the form

(10) $$\begin{array}{l} Y_{f}=X_{f}\beta_{f}+U_{f}+E_{f}+h\left(U_{s}\right), \end{array}$$

as considered by Töpner et al. (2017) and Grotzinger et al. (2019). In contrast to the Multi-BLUP, GM-BLUP only depends on the genetic components of the secondary traits.

Finally, following many other authors (e.g., Riedelsheimer et al., 2012; Xu et al., 2016) we will also compute a prediction based on the secondary traits alone, using the model

(11) $$\begin{array}{l} Y_{f}=X_{f}\beta_{f}+V_{f}^{\left(\text{sec}\right)}+E_{f}=X_{f}\beta_{f}+Y_{s}b_{s}+E_{f}, \end{array}$$

and define the MBLUP

(12) $$\begin{array}{l} {\hat{U}}_{M}={\hat{V}}_{f}^{\left(\text{sec}\right)}=Y_{s}{\hat{b}}_{s}. \end{array}$$

Again, this is to some degree a phenotypic prediction, and since the direct effects of the SNPs are ignored, the estimated effects ${\hat{b}}_{s}$ will differ from those obtained from model (7).

### 2.8. Simulations

We first compare the different methods on simulated data, with *p* = 300 secondary traits. We used existing genotypic data, from the Arabidopsis RegMap, containing 1, 307 accessions genotyped with 214, 051 SNPs (Horton et al., 2012). For each data-set we randomly selected 500 accessions, from which we randomly sampled a test set of 100 accessions. We randomly selected 1, 500 SNPs with a minor allele frequency of at least 0.3. For each data-set we first simulated direct genetic effects (*g*_*i*_) and residuals (*r*_*i*_) for each accession *i*, and the final trait values were obtained using a structural equation model, describing functional relations between traits. More specifically, for each individual *i*, the (*p* + 1) × 1 vector of trait values is defined by *y*_*i*_ = *y*_*i*_Λ + *g*_*i*_ + *r*_*i*_, Λ being the (*p* + 1) × (*p* + 1) matrix of structural coefficients. The (*k, l*)th entry of Λ contains the effect of trait *k* on trait *l*, and the vectors *g*_*i*_ and *r*_*i*_ have zero mean Gaussian distributions with covariance matrices Σ^*g*^ and Σ^*r*^, respectively. The joint distribution of all *n*(*p* + 1) trait values is then as in (1), with Σ^*u*^ = Γ^*t*^Σ^*g*^Γ and Σ^*e*^ = Γ^*t*^Σ^*r*^Γ, where Γ = (*I* − Λ)^−1^ (Gianola and Sorensen, 2004; Töpner et al., 2017; Kruijer et al., 2020).

The target trait is defined as *Y*_*f*_ = *Y*_1_ = λ(*Y*_2_ + *Y*_3_ + *Y*_4_) + *G*_1_ + *R*_1_, and we do not assume any functional relations among the secondary traits. Hence, if λ ≠ 0, there is a causal effect from *Y*_2_, *Y*_3_, and *Y*_4_ on *Y*_1_, but the algorithms under consideration do not know which of the 300 secondary traits are the actual causal ones. We consider λ values on the grid {−1, −0.5, 0, 0.5, 1}. Σ^*g*^ has diagonal elements (0.2, 0.7, …, 0.7), i.e., the variances of the direct genetic effects are 0.2 for *Y*_*f*_ and 0.7 for each of the secondary traits. The off-diagonal elements corresponding to *Y*_1_ vs. (*Y*_2_, *Y*_3_, *Y*_4_) are $\rho_{G}\sqrt{0.2\cdot 0.7}$, where we choose ρ_*G*_ ∈ {−0.5, 0, 0.5}. Similarly, Σ^*r*^ has diagonal elements 0.8 for *Y*_*f*_ and 0.3 for the secondary traits, and the off-diagonal elements between *Y*_1_ and (*Y*_2_, *Y*_3_, *Y*_4_) are $\rho_{E}\sqrt{0.8\cdot 0.3}$, with ρ_*E*_ ∈ {−0.5, 0, 0.5}. The other off-diagonal elements in Σ^*g*^ and Σ^*r*^ are zero.

For the special case λ = 0 we have Γ = *I*, Σ^*u*^ = Σ^*g*^ and Σ^*e*^ = Σ^*r*^, and *Y*_*f*_ will have a heritability of 0.2. The secondary traits will have heritability 0.7, and there is no causal effect of (*Y*_2_, *Y*_3_, *Y*_4_) on *Y*_1_. Genomic prediction for *Y*_1_ can however still benefit from the genetic correlation between these traits (which is present when ρ_*G*_ ≠ 0). When λ ≠ 0, the causal effect of (*Y*_2_ + *Y*_3_ + *Y*_4_) on *Y*_1_ will introduce additional genetic and residual covariance in Σ^*u*^ and Σ^*e*^.

For each of the 125 combinations of λ, ρ_*G*_ and ρ_*E*_ we simulate 50 data-sets; for each of them we predicted the simulated genetic effects for the test set, with the different methods.

#### 2.8.1. Benchmark

In addition to the methods described above, we evaluate a benchmark prediction, by computing (4) and (5) for the four-dimensional mixed model with *Y*_1_ − *Y*_4_, using the true (simulated) variance components.

### 2.9. Data

To test the methods on real data, we consider four data-sets with various target and secondary phenotypes. To assess accuracy, each data set was randomly split into training (70%) and a test genotypes (30%). This was repeated 160 times, and we report accuracy averaged over the 160 test sets. Because of the required computing time, only 50 test sets were analyzed for RF-BLUP with hyper-parameter-optimization (for the Arabidopsis data-sets), and 30 test-sets for the maize data (for all methods). With one exception (mentioned below), the target and secondary phenotypes were measured on different plants; therefore, all bivariate mixed models were fitted with diagonal residual covariance (i.e., diagonal Σ^*e*^ in Equations 4 and 5).

The first two data sets were measured on the *A. thaliana* HapMap population, where 36 metabolites from Fusari et al. (2017) were used as secondary phenotypes and the kinship matrix was estimated based on one million imputed SNPs (Arouisse et al., 2020). Dataset 1 contains three target traits related to biotic and abiotic stress, from Thoen et al. (2017). In dataset 2, the target is the rosette fresh weight, measured in of the experiments of Fusari et al. (2017). This is the only dataset for which the residual covariance is non-diagonal.

In the third data set, we predicted the grain yield, plant height (PH) and flowering time (FT) of 388 inbred maize lines (*Z. mays*), using 5, 760 transcripts (Azodi et al., 2020) as secondary traits. In this case, we selected for each data-set a subset of transcripts using the LASSO on the training set, following Azodi et al. (2020). In other words, the transcripts selected by LS-BLUP were also used for the other methods.

### 2.10. Data Availability

The data that support the findings of this study are available at:

https://doi.org/10.1105/tpc.19.00332 (Maize data)

https://doi.org/10.1105/tpc.17.00232 (*A. thaliana* Metabolite data)

https://doi.org/10.1111/nph.14220 (*A. thaliana* Phenotypes)

https://doi.org/10.1111/tpj.14659 (*A. thaliana* SNP data)

All data-sets (except the maize transcriptomics) are included in an Rdata file available at: https://figshare.com/s/5d01062711ce33bb327e.

### 2.11. Software and Computing Time

The required computing time is mainly driven by the complexity of fitting either a bivariate mixed model with a single relatedness matrix, or univariate mixed models with either one or two relatedness matrices. For the datasets considered here, each bivariate mixed model took between 20 and 50 s to fit, the univariate mixed models taking at most a few seconds. For complexity as function of *n* and *p* we refer to Zhou and Stephens (2014).

R-code for all methods is available at https://figshare.com/s/5d01062711ce33bb327e, where we mostly relied on asreml-R (Butler et al., 2009). Several open source alternatives are however available; in particular sommer (Covarrubias-Pazaran, 2016) for bivariate mixed models, and gaston for univariate mixed models. Using gaston's lmm.diago.likelihood function, the (univariate) GBLUP for large numbers of traits can be computed in only a few seconds, which is useful for the GM-BLUP method. For the dimension reduction in LS- and RF-BLUP we used the R-packages glmnet (Friedman et al., 2010), caret (https://cran.r-project.org/package=caret), and randomForest (Liaw and Wiener, 2002). For the maize data, LASSO and random-forest regression were performed in python, using the scikit-learn packages.

## 3. Results

### 3.1. Simulations

**Figure 2:**
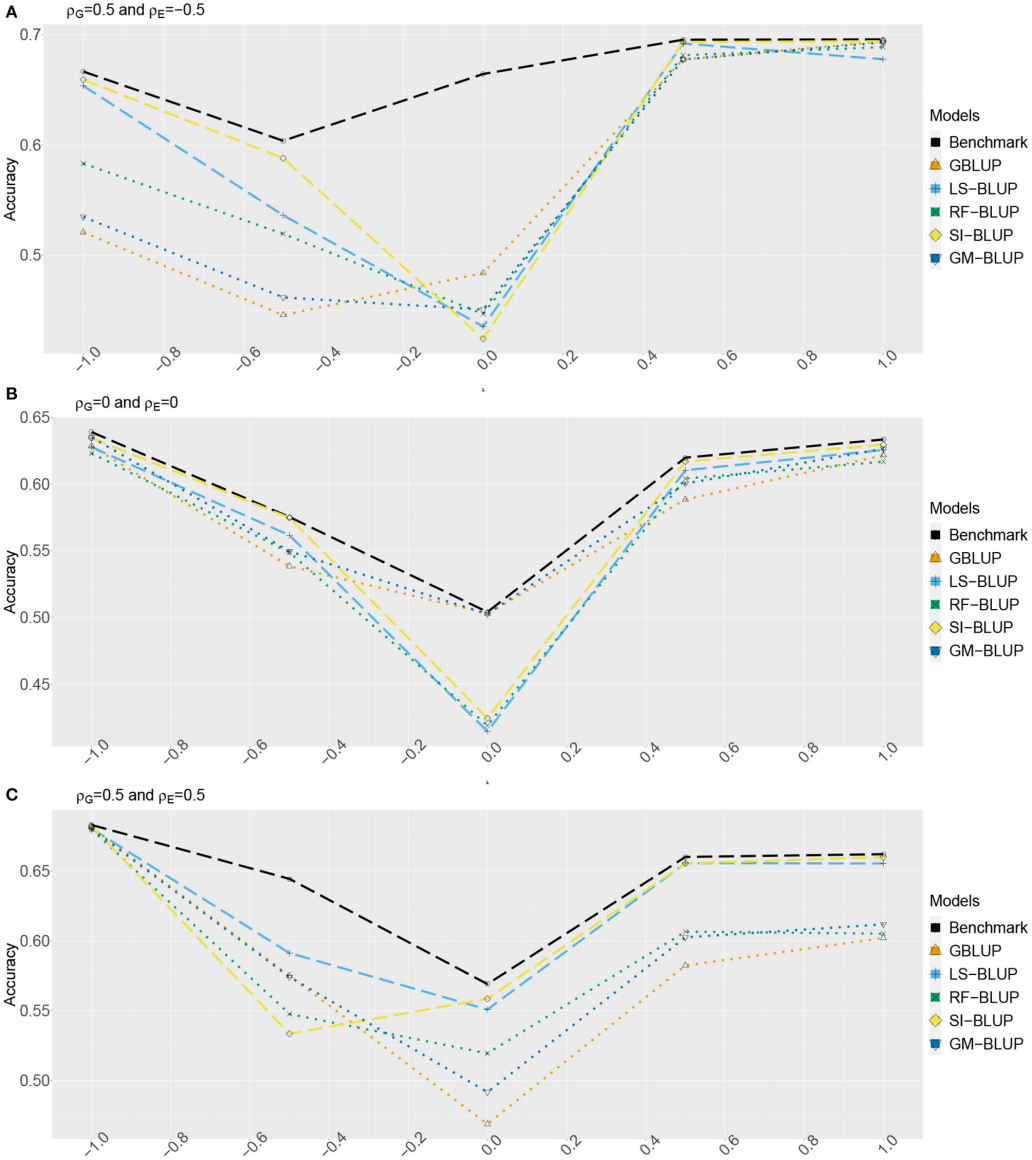
Accuracy of genomic prediction methods in scenario 1, which for each value of λ is estimated from 50 simulated data-sets (standard errors between 0.011 and 0.042). “GBLUP” is the univariate GBLUP, and the benchmark is the multivariate GBLUP based on *Y*_1_, …, *Y*_4_, using the true (simulated) values of the variance components (see section 2.8.1). Acronyms of the other methods are given in section 2; they use all secondary traits (*Y*_2_, …, *Y*_301_), without knowledge of (*Y*_2_, *Y*_3_, *Y*_4_) being causal. λ is the size of the causal effect of (*Y*_2_, *Y*_3_, *Y*_4_) on *Y*_1_. ρ_*G*_ is the correlation between the direct genetic effects on *Y*_1_, …, *Y*_4_; similarly, ρ_*E*_ is the correlation between the non-genetic effects. The total genetic correlation is function of λ and ρ_*G*_. **(A)** ρ_*G*_ = 0.5, ρ_*E*_ = −0.5, **(B)** ρ_*G*_ = 0, ρ_*E*_ = 0, and **(C)** ρ_*G*_ = 0.5, ρ_*E*_ = 0.5.

**Figure 3:**
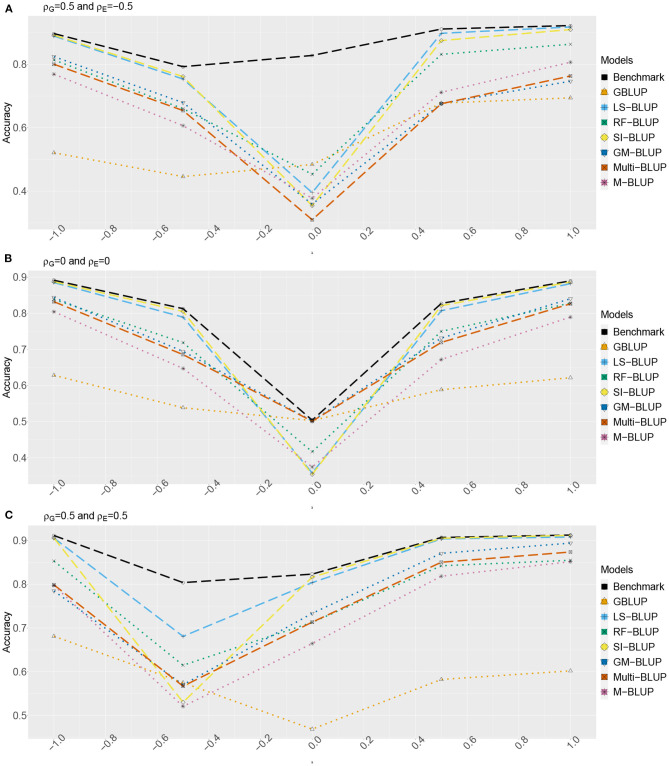
Accuracy of genomic prediction methods in scenario 2, which for each value of λ is estimated from 50 simulated data-sets (standard errors between 0.014 and 0.051). “GBLUP” is the univariate GBLUP, and the benchmark is the multivariate GBLUP based on *Y*_1_, …, *Y*_4_, using the true (simulated) values of the variance components (see section 2.8.1). Acronyms of the other methods are given in section 2; they use all secondary traits (*Y*_2_, …, *Y*_301_), without knowledge of (*Y*_2_, *Y*_3_, *Y*_4_) being causal. λ is the size of the causal effect of (*Y*_2_, *Y*_3_, *Y*_4_) on *Y*_1_. ρ_*G*_ is the correlation between the direct genetic effects on *Y*_1_, …, *Y*_4_; similarly, ρ_*E*_ is the correlation between the non-genetic effects. The total genetic correlation is function of λ and ρ_*G*_. **(A)** ρ_*G*_ = 0.5, ρ_*E*_ = −0.5, **(B)** ρ_*G*_ = 0, ρ_*E*_ = 0, and **(C)** ρ_*G*_ = 0.5, ρ_*E*_ = 0.5.

Figures 2, 3 show the estimated accuracy as function of λ, i.e., the size of the causal effects of *Y*_2_, *Y*_3_, and *Y*_4_ on the target trait *Y*_*f*_ (i.e., *Y*_1_). We focus on three cases, with different values for the correlations between the direct genetic effects on *Y*_1_, …, *Y*_4_, as well as the corresponding residuals (see section 2): (A) ρ_*G*_ = 0.5 and ρ_*E*_ = −0.5, (B) ρ_*G*_ = ρ_*E*_ = 0, and (C) ρ_*G*_ = 0.5 and ρ_*E*_ = 0.5. In scenario 1 (Figure 2) as well as scenario 2 (Figure 3), accuracies are generally higher when λ moves away from zero. This is expected, as the total genetic variance and heritability increase due to the causal effect, especially when ρ_*G*_ and λ have the same sign. When they have opposite sign, the lowest accuracy can occur at an intermediate value of λ [e.g., at λ = −0.5 in case of (A)].

The multi-trait benchmark with perfect information on the genetic and residual covariance between the target trait *Y*_*f*_ and secondary traits *Y*_2_, *Y*_3_, and *Y*_4_ always outperforms univariate GBLUP, except when ρ_*G*_ = λ = 0, in which case accuracies are equal. When ρ_*G*_ ≠ 0, the benchmark always benefits from the genetic correlations between the target trait and the secondary traits, even if the latter do not have a causal effect on *Y*_*f*_.

The accuracy of univariate GBLUP varied between *r* = 0.44 and *r* = 0.70, while the benchmark had accuracy between 0.50 − 0.70 (scenario 1) and 0.50 − 0.92 (scenario 2). The difference between scenario 2 (secondary traits observed on the test set) and scenario 1 (secondary traits only observed on the training set) was bigger for large values of |λ|. This is because for large |λ|, the total genetic correlation (which is also a function of ρ_*G*_) between *Y*_*f*_ and the causal secondary traits (*Y*_2_, *Y*_3_, and *Y*_4_) is larger.

In absence of a causal effect *Y*_*s*_ → *Y*_*f*_ (λ = 0) and residual genetic and residual correlations having opposite sign (case A), our simulation setup appeared to be too challenging, and none of the methods performed better than univariate GBLUP. Something similar occurred in case C, for λ = −0.5. On the positive side, for large values of |λ|, both SI-BLUP and LS-BLUP have near-benchmark accuracy, where the latter did not rely on plot-level observations. In scenario 2, RF-BLUP appeared to be an interesting alternative, with somewhat lower accuracy on the extreme sides, but relatively good performance at unfavorable values of λ.

Prediction based on the secondary traits only (M-BLUP; only available in scenario 2) is generally one of the least successful. The multi-kernel methods (Multi-BLUP and GM-BLUP) are somewhere in between, GM-BLUP often having an accuracy similar to that of RF-BLUP. GM-BLUP appears to be slightly better than Multi-BLUP, but in most cases the difference is smaller than the standard errors of the accuracy estimates.

### 3.2. Arabidopsis and Maize Data

Tables 1, 2 contain the accuracies for datasets 1–4 described above, averaged over randomly sampled test sets (see section 2). Because the original individual plant (or plot) data were not available, we could not compute the SI-BLUP here.

**Table 1:** Prediction accuracy in scenario 1, for various target and secondary traits in Maize and Arabidopsis.

**Data sets**	**Target trait**	**Secondary phenotypes**	**GBLUP**	**GM-BLUP**	**LS-BLUP**	**RF-BLUP**	RF-BLUP*
1	Number of spreading lesions	Metabolites	0.23	0.22	0.20	0.21	0.21
	under fungus stress						
	Fresh weight of the rosette	Metabolites	0.03	0.00	0.07	0.09	0.09
	under Salt_5 stress						
	Number of spreading lesions	Metabolites	0.19	0.18	0.16	0.16	0.15
	under Drought_and_fungus stress						
	Number of damaged leaves and	Metabolites	0.10	0.09	0.06	0.10	0.10
	feeding sites under Caterpillar_3 stress						
2	Fresh weight	Metabolites	0.30	0.30	0.29	0.30	0.30
3	Flowering time (FT) [4]	Transcripts	0.54	0.55	0.55	0.53	0.55
	Plant height (PH)	Transcripts	0.54	0.55	0.55	0.53	0.51
	Yield	Transcripts + FT+PH	0.53	0.53	0.54	0.52	0.52
	Yield	Transcripts	0.55	0.55	0.55	0.55	0.55

Acronyms of the methods are as in Figures 2, 3. For RF-BLUP*, we used the randomForest package with the default settings; for RF-BLUP, hyper-parameters were optimized using the caret package (data-sets 1 and 2) or scikit-learn (data-set 3). For data-sets 1 and 2, reported accuracies are averages over 160 test sets (standard errors between 0.006 and 0.007), except for RF-BLUP, where 50 sets were used (SE between 0.010 and 0.014). In dataset 3, 30 test sets were used for all methods (SE between 0.006 and 0.03).

**Table 2:** Prediction accuracy in scenario 2, for various target and secondary traits in Maize and Arabidopsis.

**Data sets**	**Target trait**	**Secondary phenotypes**	**GBLUP**	**M-BLUP**	**Multi-BLUP**	**GM-BLUP**	**LS-BLUP**	**RF-BLUP**	RF-BLUP*
1	Number of spreading lesions	Metabolites	0.23	−0.04	0.21	0.22	0.31	0.28	0.28
	under fungus stress								
	Fresh weight of the rosette	Metabolites	0.03	0.09	0.08	0.07	0.23	0.20	0.19
	under Salt_5 stress								
	Number of spreading lesions	Metabolites	0.19	−0.02	0.16	0.17	0.27	0.25	0.23
	under Drought_and_fungus stress								
	Number of damaged leaves and	Metabolites	0.10	0.05	0.06	0.07	0.14	0.12	0.11
	feeding sites under Caterpillar_3 stress								
2	Fresh weight	Metabolites	0.30	0.00	0.29	0.30	0.32	0.30	0.28
3	Flowering time (FT) [4]	Transcripts	0.55	0.54	0.55	0.55	0.66	0.65	0.54
	Plant height (PH)	Transcripts	0.54	0.53	0.54	0.55	0.66	0.64	0.53
	Yield	Transcripts + FT+PH	0.53	0.49	0.50	0.52	0.72	0.71	0.49
	Yield	Transcripts	0.55	0.52	0.53	0.54	0.64	0.65	0.51

Acronyms of the methods are as in Figures 2, 3. For RF-BLUP*, we used the randomForest package with the default settings; for RF-BLUP, hyper-parameters were optimized using the caret package (data-sets 1 and 2) or scikit-learn (data-set 3). For data-sets 1 and 2, reported accuracies are averages over 160 test sets (standard errors between 0.006 and 0.012), except for RF-BLUP, where 50 sets were used (SE between 0.010 and 0.014). In dataset 3, 30 test sets were used for all methods (SE between 0.006 and 0.03).

In scenario 1 (Table 1), none of the multi-trait methods performed consistently better than univariate GBLUP. For the second trait in data-set 1 (Salt5), RF-BLUP had accuracy 0.09, vs. 0.03 for univariate GBLUP; the latter had highest accuracy for the first and third trait in dataset 1 (fungus, and drought and fungus stress combined).

The remainder of this section we focus on scenario 2 (Table 2), in which there were more substantial differences among methods. For all datasets, methods based on multiple relatedness matrices (Multi-BLUP and GM-BLUP) had accuracies similar to single-trait GBLUP. As in the simulations, GM-BLUP gave only a minor (if any) improvement over Multi-BLUP. The approaches based on dimension reduction of the secondary traits (LS-BLUP and RF-BLUP) appeared to give a substantial improvement over univariate GBLUP, e.g., from *r* = 0.03 to *r* = 0.23 (LS-BLUP) for the Salt5 trait in data-set 1, or from *r* = 0.55 to *r* = 0.65 (RF-BLUP) for Maize yield in data-set 3, with transcriptomics as secondary traits.

LS-BLUP had the highest accuracy in all Arabidopsis datasets, with a small but consistent improvement over RF-BLUP (0.02–0.03 higher), also when optimized with the caret/scikit-learn packages. This hyperparameter optimization appeared to be rather important for the Maize data; using the default settings from the randomForest package (as in the simulations), accuracy was considerably lower (for yield and the transcripts for example, *r* = 0.65 vs. *r* = 0.51).

For the maize data, RF/LS-BLUP improved accuracy for yield from around 0.64 − 0.65 to 0.71 − 72 when plant height and flowering time were included as secondary phenotypes, together with the transcriptome data. None of the other methods could exploit the additional data, and accuracies were similar to those obtained with the transcripts alone. Prediction based on the secondary traits alone (M-BLUP) had around zero accuracy in all Arabidopsis data-sets, but *r* = 0.49−0.54 for the maize data, similar to GBLUP and multi-BLUP.

## 4. Discussion

Given the importance of genomic selection in plant breeding and the rapid development of phenotyping technology, it becomes increasingly important to know if and how the availability of additional phenotypic traits can improve prediction accuracy for a target trait. Here we proposed new methods to incorporate large numbers of such additional traits in genomic prediction, and compared these to existing methods, in simulated and real data. In many of the simulated data-sets, some of our methods indeed greatly improved univariate genomic prediction. In these cases, the accuracy was often close to that of penalized selection indices, without requiring plot-level data. In other cases, none of the methods did very much better than univariate prediction, while the multi-trait benchmark indicated that there is in fact scope for improvement. This happens especially when genetic and residual correlation have opposite sign. Moreover, our study indicates that current methods do not perform well when the secondary traits are available only on the training set (i.e., in scenario 1): while there was often some improvement in many of the simulations, accuracy in scenario 1 was hardly improved for any of the real data-sets.

While scenario 1 is probably most common, scenario 2 (secondary traits being also observed for the test set) may arise in a number of applications. In particular, it has become increasingly common to screen large collections for metabolites or other types of -omics data, and scenario 2 may also arise in a biomedical context when biomarkers could be used to predict disease. Our results for various stress traits in Arabidopsis showed that metabolites can indeed improve accuracy, even if they were measured in a different study. While Multi-BLUP and the LS- and RF-BLUP require balanced data, the GM-BLUP is more flexible, and can also handle an intermediate scenario where only some of the secondary traits are measured for all (or some of) the test genotypes.

Except SI-BLUP, all methods implicitly assume a causal relationship between the secondary traits and the target trait. In our simulations, accuracy was indeed suboptimal when this relationship was weak or absent. However, in these cases the SI-BLUP often performed poorly as well. The accuracy of LS-BLUP and RF-BLUP may be improved if one could successfully address the following two artifacts. First, the dimension reduction and genomic prediction should ideally be carried out on different subsets of the training set. In the populations we considered here, this however led to poor estimation of variance components and lower accuracies, because of the relatively small population size. We therefore used the whole training set for both dimension reduction and genomic prediction. The advantage of a larger training set seems to outweigh the incurred overfitting, but this may be different for larger populations, in which case sub-sampling strategies like bootstrap aggregation (bagging) might be useful. Second, specifically for LS-BLUP, the cross-validation in the first (dimension reduction) step appears to select too many variables. Often, this may still result in an accurate prediction Ŷ_*s*_ on the training set, but for the prediction of breeding values on the test set that leads to overfitting. The methodology implemented in the hdi-package (Dezeure et al., 2015) might resolve this issue, by first assessing significance of secondary traits. Such improvements should at least guarantee an accuracy that is never (much) below that of univariate GBLUP. Finally, a remaining limitation of RF-BLUP and LS-BLUP is that the dimension reduction relies on phenotypic rather than genetic values, which is likely to stay sub-optimal in case genetic and residual correlations have opposite sign.

We attempted to improve existing multi-kernel methods with our GM-BLUP approach, replacing secondary traits by their genomic predictions. Unfortunately, this led to only minor improvements. In case secondary traits have high heritability, there is little shrinkage and genomic predictions and trait values are highly correlated, leading to similar accuracies. In case secondary traits have lower heritabilities, the methods may potentially differ more, but at the same time, in such a scenario there is much less scope for improvement with multi-trait methods in the first place. Both Multi-BLUP and GM-BLUP were often less accurate than competing methods. To some extent this may be explained by the absence of variable selection, or, compared to RF-BLUP, the assumed linearity. Nonetheless, GM-BLUP extended the use of Multi-BLUP to scenario 1, without ever being less accurate.

For the case of a single secondary trait, Runcie and Cheng (2019) studied the bias in accuracy estimates, when these are based on the correlation with the observed phenotype, rather than with the (unobserved) genetic effect. This can become problematic when traits are measured on the same plants, in which case the amount of bias is likely to vary among methods, in particular when residual correlations between the target and secondary traits are large. For the Arabisopsis and maize data considered here, the bias should be constant, as all target and secondary traits were measured on different plants. No bias occurred for the simulated data, where we used the true genetic values to assess accuracy. Nevertheless, further work is needed to extend the methods presented here with reliable estimates of accuracy, also in the case of traits measured on the same plants. For the LS-BLUP, RF-BLUP and SI-BLUP, the parametric and semi-parametric accuracy estimates of Runcie and Cheng (2019) can in principle be computed, since all these methods reduce the dimension of the secondary traits to one. This would however require the sample-splitting or bagging schemes mentioned above, and it is an open question how the different accuracy estimates should be aggregated.

Statistical methods for high-dimensional data often benefit from initial screening, for example by removing variables with very low marginal correlation (see e.g., Fan and Lv, 2008). In the present context, screening should be based on heritability and genetic correlation with the target trait. This is however difficult for several reasons. First, as pointed out before, reliable estimates of these correlations require plot-level data, at least for the population sizes considered here. Moreover, bivariate mixed models need to be fitted for each secondary trait, increasing computation time. A more fundamental problem is that even if accurate estimates were available, it would be difficult to formulate an appropriate criterion and threshold. The well-known criterion for a single secondary trait (whose heritability times the squared genetic correlation with the target trait should exceed the heritability of the latter) cannot directly be generalized. For example, in one of our simulation settings (i.e., with λ = 0 and ρ_*G*_ = 0.5), each of the three relevant secondary traits (*Y*_2_, *Y*_3_, *Y*_4_) has heritability 0.7, the heritability of the target trait being 0.2. Consequently, we have $0.7\times \rho_{G}^{2}<0.2$ for each secondary trait individually, while at the same time genomic prediction using a mixed model for *Y*_1_ − *Y*_4_ is more accurate than with a mixed model for *Y*_1_ alone.

More generally, the methods presented here could be extended in several ways. First, for all of them, prediction relies on the GBLUP: either bivariate GBLUP, or univariate GBLUP extended with additional relatedness matrices. This corresponds to a Gaussian prior on the marker effects, which could be generalized to a mixture of Gaussians and a point mass at 0, as for example in Bayes-R (Moser et al., 2015). Another extension would be the prediction of sensitivities to environmental covariates, which could then be used to predict new environments, as in Millet et al. (2019). In the LS- and RF-BLUP methods, a wider range of prediction methods could be considered to achieve the dimension reduction, such as elastic nets or gradient tree boosting. Ideally, this reduction is driven by genetic rather than phenotypic effects, and the dimension should not necessarily be reduced to one (like we did here), but to a data-driven number. Finally, it would be of interest to relax the linearity assumption on which most methods (except RF-BLUP) rely. Deep learning with feedforward or convolutional neural networks seems of particular interest here, especially for the relationship between target and secondary traits.

## Author Contributions

BA performed the research. WK, BA, and FE designed the research. BA and WK wrote the paper, with input from TT and FE. All authors contributed to the article and approved the submitted version.

## Conflict of Interest

The authors declare that the research was conducted in the absence of any commercial or financial relationships that could be construed as a potential conflict of interest.
